# A Case of Hypoglycemia With Concomitant Use of a Sulfonylurea and Clopidogrel

**DOI:** 10.7759/cureus.26306

**Published:** 2022-06-24

**Authors:** Brent Ruiz, Karina G Romo, Jeremy Teruel, Jared Dendy, Qasim Z Iqbal

**Affiliations:** 1 Internal Medicine, Ochsner Clinic Foundation, New Orleans, USA; 2 Endocrinology, Diabetes and Metabolism, Ochsner Clinic Foundation, New Orleans, USA

**Keywords:** type 2 diabetes mellitus, peripheral arterial diseases, plavix, glimepiride, hypoglycemia

## Abstract

Hypoglycemia secondary to sulfonylureas and clopidogrel have been independently described in the literature. However, there has been minimal investigation into the risk of clopidogrel-induced hypoglycemia in the setting of long-term or concomitant sulfonylurea use in patients with Type 2 diabetes mellitus. We present a case of a patient with diabetes well managed on glimepiride (second-generation sulfonylurea) for more than 10 years who presented with an episode of hypoglycemia shortly after initiation of clopidogrel for peripheral vascular disease.

## Introduction

While hypoglycemia secondary to sulfonylureas and clopidogrel has been independently described, there has been little investigation into the risk of clopidogrel-induced hypoglycemia in the setting of long-term or concomitant sulfonylurea use in Type 2 diabetes mellitus (T2DM). The cytochrome P450 family 2 subfamily C member 9 (CYP2C9) enzyme plays a role in both the metabolism of clopidogrel and sulfonylureas: clopidogrel's sulfhydryl (thiol) metabolite is catalyzed in part by CYP2C9 enzyme, likewise, sulfonylureas are extensively metabolized by CYP2C9 into a metabolically active substrate [1,2]. This has led to concerns regarding the concomitant use of the two medications; not only is there a theoretical increased risk of hypoglycemia via competitive inhibition but also, studies have demonstrated lower efficacy of clopidogrel on platelet function when taken simultaneously with a sulfonylurea. In fact, given the nature of the situations in which clopidogrel is necessary (i.e., dual antiplatelet therapy (DAPT) post percutaneous coronary intervention (PCI) and stent placement) and the severity of adverse effects should DAPT fail, said studies recommend the use of ticagrelor as a substitute for clopidogrel in patients requiring DAPT who are also on a sulfonylurea [3]. Not only may co-prescribing clopidogrel and sulfonylureas lead to reduced efficacy of clopidogrel on platelet function, but also, if a significant risk of hypoglycemia exists, then patients who require sulfonylureas should be considered for ticagrelor rather than clopidogrel therapy when necessary. The following highlights a case of a patient with T2DM and chronic use of glimepiride, a second-generation sulfonylurea, who presented with new hypoglycemia shortly after initiating clopidogrel.

## Case presentation

An 86-year-old Latino man with a pertinent medical history of T2DM on glimepiride, ischemic cardiomyopathy s/p implantable cardioverter defibrillator, hypertension, dyslipidemia, and severe peripheral artery disease (PAD) presented with an acute onset of symptomatic hypoglycemia five days after initiation of clopidogrel 75 mg daily by his outpatient vascular surgeon for PAD. His symptoms included persistent confusion, irritability, and fatigue. He was found to have a fasting blood glucose (BG) of 67 mg/dl and then 31 mg/dl on repeat by Emergency Medical Services (EMS). His BG was responsive to glucose tablets. There were no other complaints including no recent weight changes or changes in appetite or eating pattern (no missed meals). He had a planned outpatient lower extremity (LE) angiography for PAD. He had no recent changes to his medications aside from the recent initiation of clopidogrel. His home medications prior to admission included the use of glimepiride 4 mg daily, which he had been taking for 10 years without any prior episode of symptomatic hypoglycemia. On admission, he was afebrile and hemodynamically stable. His physical examination revealed no focal neurologic deficit and was unremarkable aside from bilateral LE wounds and + 1 pedal pulses. His admission hemoglobin A1c (HbA1c) was 9.0%, morning 8 am cortisol was within normal limits, and his insulin autoimmune antibody was negative. Glimepiride was discontinued on admission. The patient underwent a popliteal artery stenting while an inpatient. A decision was made to discontinue clopidogrel and start ticagrelor after discussion with the primary and vascular surgery teams. This led to the resolution of hypoglycemia after 48 hours. The interaction of clopidogrel and glimepiride was diagnosed by the temporal association in the occurrence of hypoglycemia and hypoglycemic symptoms with clopidogrel initiation.

## Discussion

In people without diabetes, a serum glucose concentration <70 mg/dL is the threshold at which the body initiates the neuroendocrine response to decreasing serum glucose levels. Patients with diabetes are unable to mount an appropriate counterregulatory hormone response and have a diminished autonomic response (Table 1) to hypoglycemia. Hypoglycemia unawareness is defined as the onset of neuroglycopenic symptoms before the start of neurogenic symptoms in level 2 hypoglycemia. Major risk factors in hypoglycemia unawareness include duration of diabetes, history of recent/recurrent hypoglycemic episodes, and intense glycemic control. Patients experiencing this phenomenon should be reassessed along with the review of current medications and glycemic targets. Avoiding hypoglycemia for several weeks can result in a partial reversal of hypoglycemia unawareness. Frequent glucose monitoring is essential in the detection and avoidance of hypoglycemia episodes that may result in harm to self or others.

**Table 1:** Common Autonomic and Neuroglycopenic Symptoms of Acute Hypoglycemia.

Autonomic	Neuroglycopenic
Palpitations	Speech Difficulty
Anxiety	Incoordination
Tremor	Dizziness
Paresthesias	Altered Mental Status
Diaphoresis	Seizures
Hunger	Coma

Although rare, clopidogrel-induced hypoglycemia has been associated with insulin autoimmune syndrome (IAS), most frequently cited in Japanese populations. It is a form of hyperinsulinemic hypoglycemia with elevated anti-insulin levels. In published case reports, IAS has been detected in the setting of new-onset hypoglycemia after initiation of sulfhydryl group medications (i.e., clopidogrel) [4]. In this case, IAS was considered initially; however, the suspicion for IAS lowered given the negative insulin autoimmune antibody test. 

Among oral anti glycemic medications, sulfonylureas are well tolerated but carry an increased risk of hypoglycemia. In patients with T2DM, hypoglycemia may occur due to pharmacological agents used to achieve adequate glycemic control. Furthermore, the risk for iatrogenic hypoglycemia remains an obstacle to safely achieving tighter glycemic targets. Elderly patients and those with comorbidities (i.e., cardiovascular disease, renal insufficiency, cognitive and functional disorders, stroke, polypharmacy, cancer, etc.) are especially vulnerable to severe hypoglycemic episodes. The severity of hypoglycemia is based on symptoms and/or serum glucose levels. The American Diabetes Association categorizes hypoglycemia into three levels. Level 1 is defined as a serum glucose concentration <70 mg/dL but ≥54 mg/dL and is considered clinically important regardless of the presence or absence of acute neurogenic symptoms (i.e., irritability, palpitations, diaphoresis, tremor, etc.). Level 2 is defined as a serum glucose concentration < 54 mg/dL; it is at this threshold that neuroglycopenic symptoms (i.e., confusion, seizures, coma, etc.) are observed. Level 3 is defined as a severe event characterized by altered mental status or physical impairment requiring intervention by another person for reversal.

Cardiovascular disease is the leading cause of death in individuals with diabetes mellitus. The link between hyperglycemia and cardiovascular disease has been well established [5]. However, observational studies suggest hypoglycemia increases the risk for adverse cardiovascular events and all-cause mortality in patients with diabetes. In patients with diabetes and pre-existing vascular disease, acute and chronic hypoglycemia may result in myocardial infarction and/or ischemic stroke. Snell at el. theorized that recurrent hypoglycemia may pose the greatest cardiovascular disease risk among patients who have already sustained at least a decade of vascular damage due to diabetes. Effects of hypoglycemia on the heart include lengthening of the QTc interval and arrhythmias (i.e., bradycardia) that may contribute to sudden cardiac death.

## Conclusions

Given the increased risk of adverse cardiovascular events in patients with diabetes and hypoglycemic events, it is imperative that we identify drug interactions that may increase the risk of hypoglycemia. This report highlights a case of hypoglycemia with concomitant use of a sulfonylurea (glimepiride) and clopidogrel. There is a potential CYP2C9 mediated interaction, i.e., competitive inhibition, between clopidogrel and glimepiride. The interaction not only may reduce clopidogrel's efficacy on platelet function but may also exaggerate the pharmacodynamic effects of sulfonylureas and increase the risk for hypoglycemic events in T2DM patients when co-prescribed. If anti-platelets are indicated in patients on sulfonylureas, the use of non-sulfhydryl anti-platelets such as ticagrelor should be considered instead of clopidogrel. More research is needed to review hypoglycemia frequency and trends in patients concurrently prescribed clopidogrel and sulfonylureas to establish if a significant risk of hypoglycemia exists. 
